# Association of *CETP* Gene Polymorphisms and Haplotypes with Cardiovascular Risk

**DOI:** 10.3390/ijms241210281

**Published:** 2023-06-17

**Authors:** Peter Piko, Tibor Jenei, Zsigmond Kosa, Janos Sandor, Nora Kovacs, Ildiko Seres, Gyorgy Paragh, Roza Adany

**Affiliations:** 1 Department of Public Health and Epidemiology, Faculty of Medicine, University of Debrecen, 4032 Debrecen, Hungary; piko.peter@med.unideb.hu (P.P.); jenei.tibor@med.unideb.hu (T.J.); sandor.janos@med.unideb.hu (J.S.); kovacs.nora@med.unideb.hu (N.K.); 2 National Laboratory for Health Security, Center for Epidemiology and Surveillance, Semmelweis University, 1089 Budapest, Hungary; 3 Department of Health Methodology and Public Health, Faculty of Health, University of Debrecen, 4400 Nyíregyhza, Hungary; kosa.zsigmond@foh.unideb.hu; 4 ELKH-DE Public Health Research Group, Department of Public Health and Epidemiology, Faculty of Medicine, University of Debrecen, 4028 Debrecen, Hungary; 5 Institute of Internal Medicine, Faculty of Medicine, University of Debrecen, 4032 Debrecen, Hungary; seres@belklinika.com (I.S.); paragh@internal.med.unideb.hu (G.P.); 6 Department of Public Health, Semmelweis University, 1089 Budapest, Hungary

**Keywords:** cholesteryl ester transfer protein, single-nucleotide polymorphism, haplotype, high-density lipoprotein cholesterol, HDL subfraction profile, Systematic Coronary Risk Evaluation, Framingham Risk Score

## Abstract

Cholesteryl ester transfer protein (*CETP*) is known to influence HDL-C levels, potentially altering the profile of HDL subfractions and consequently cardiovascular risk (CVR). This study aimed to investigate the effect of five single-nucleotide polymorphisms (SNPs; rs1532624, rs5882, rs708272, rs7499892, and rs9989419) and their haplotypes (H) in the *CETP* gene on 10-year CVR estimated by the Systematic Coronary Risk Evaluation (SCORE), the Framingham Risk Score for Coronary Heart Disease (FRS_CHD_) and Cardiovascular Disease (FRS_CVD_) algorithms. Adjusted linear and logistic regression analyses were used to investigate the association of SNPs and 10 haplotypes (H1–H10) on 368 samples from the Hungarian general and Roma populations. The T allele of rs7499892 showed a significant association with increased CVR estimated by FRS. H5, H7, and H8 showed a significant association with increased CVR based on at least one of the algorithms. The impact of H5 was due to its effect on TG and HDL-C levels, while H7 showed a significant association with FRS_CHD_ and H8 with FRS_CVD_ mediated by a mechanism affecting neither TG nor HDL-C levels. Our results suggest that polymorphisms in the *CETP* gene may have a significant effect on CVR and that this is not mediated exclusively by their effect on TG and HDL-C levels but also by presently unknown mechanisms.

## 1. Introduction

Cardiovascular disease (CVD) is the number one cause of death worldwide, with more than 20 million deaths in 2021 [1,2]. It is well known for a long time that the onset of CVDs can be delayed or prevented by reducing the negative impact of environmental and lifestyle risk factors (such as smoking, unhealthy diet, harmful use of alcohol, and physical inactivity) [3]. In addition to these, non-modifiable ones (such as age, sex, ethnicity, and individual genetic background) [4] also play an important role in the development and progression of CVDs.

Special algorithms can be used to estimate the risk of developing cardiovascular events (CVE) within a certain period of time. These algorithms use a combination of modifiable and non-modifiable factors [5] to estimate the probability of a CVE. The Framingham Risk Score [6] (FRS) and the Systematic Coronary Risk Evaluation [7] (SCORE), which estimate the risk of a CVE within 10 years, are the best-known and most widely used ones. Both consider high-density lipoprotein cholesterol (HDL-C) levels among many other factors (such as age, sex, ethnicity, total cholesterol, blood pressure, etc.) when estimating cardiovascular risk (CVR).

Epidemiological studies have consistently shown that plasma concentration of HDL-C is inversely associated with the development of atherosclerotic vascular disease [8,9]. However, Mendelian randomisation studies have failed to demonstrate a causal relationship between HDL-C levels and the occurrence of CVE [10]. There is a U-shaped relationship between HDL-C levels and CVDs [11], which can be explained by the fact that HDL-C delays the development of atherosclerotic lesions by several mechanisms [12], the most important of which is reverse cholesterol transport (RCT-removal of cholesterol from peripheral tissues as the arterial wall and transport to the liver for redistribution or excretion in bile and faeces) [13]. 

HDL is not a homogenous plasma lipoprotein fraction, it can be divided into subfractions that differ in size, density, and components and have different associations with the development of CVDs [14,15,16,17]. HDL subfractions can be classified into three main subclasses: large- (HDL-L), intermediate- (HDL-I), and small-HDL (HDL-S). The HDL subfraction profile of an individual strongly correlates with the degree of CVR; higher concentrations of large HDL (HDL_1–3_ subfractions) are associated with a lower [16], whereas those of small HDL (HDL_8–10_ subfractions) are associated with an increased risk [17,18].

To a great extent, an individual’s HDL-C level is (approximately 50% of the variability) genetically determined [19]. Cholesteryl ester transfer protein (*CETP*) gene has a prominent role as the CETP protein is involved in the RCT process and—although it is the subject of intense speculations and discussions—considered to be an effective target of interventions for CVD prevention [20]. The pathway targeted to inhibit the RCT process is the exchange of esterified cholesterol for triacylglycerol (TG) from HDL-C to low-density lipoproteins (LDL) and very-low-density lipoproteins (VLDL) via CETP [21]. A possible way to increase HDL-C levels (and potentially reduce CVR) is to inhibit CETP. Inhibition of CETP in humans increases the concentration of cholesterol in the potentially protective HDL subfractions while reducing it in harmful non-HDL ones [22]. 

Through its role in cholesteryl ester (CE) triacylglycerol exchange, which plays a role in the development of atherosclerosis and other CVDs, CETP exerts pro- and anti-atherogenic activity [23]. The high activity of the *CETP* gene may have a pro-atherogenic effect by reducing circulating HDL-C levels and participating in the CE accumulation of atherogenic apoB-containing lipoproteins (LDL and VLDL) [24]. High plasma concentrations of CETP have been associated with faster progression of coronary atherosclerosis [25] and increased carotid intima-media thickness [26,27]. 

The results of our previous studies have confirmed that single-nucleotide polymorphisms (SNPs) [28,29] and their haplotype combinations [30] in the *CETP* gene significantly influence lipid profiles through their effects on HDL-C and TG levels. It has also been shown that the HDL subfraction profile (especially the representation of HDL-L and -I subclass) is associated with estimated cardiovascular risk [31]. 

However, it is currently unknown how these SNPs and haplotypes (H), which were the subjects of our previous studies, are associated with the CVR and how they influence the HDL subfraction profile.

Therefore, our present study aims to investigate the association of five SNPs (rs1532624, rs5882, rs708272, rs7499892, and rs9989419) and their H in the *CETP* gene, identified in our previous studies, on the CVR estimated by SCORE and FRS, as well as to analyse the effects of ones significantly associated with CVR on HDL subfraction profile.

## 2. Results

### 2.1. Characteristics of Study Subpopulations, Results of Hardy–Weinberg Equilibrium (HWE), Linkage Disequilibrium (LD), and Allele and Haplotype Frequencies by Analyses

The control (subgroup with normal HDL-C, TG, and LDL levels) and case (with reduced HDL-C levels) groups used in the SCORE analyses showed a significant difference in mean body-mass index (BMI; control: 25.14 kg/m^2^ vs. case: 29.29 kg/m^2^, *p* < 0.001), total cholesterol levels (TC; control: 4.59 mmol/L vs. 5.05 mmol/L, *p* = 0.004), triacylglycerol levels (TG; control: 0.97 mmol/L vs. case: 2.08 mmol/L, *p* < 0.001), and HDL-C levels (control: 1.62 mmol/L vs. case: 1.01 mmol/L, *p* < 0.001), as well as the proportion of women (control: 48.08% vs. case: 75.81%, *p* < 0.001).

The control and case groups used in the FRSs analyses showed a significant difference in mean age (control: 48.12 years vs. case: 44.29 years, *p* = 0.004), BMI (control: 24.78 kg/m^2^ vs. case: 29.07 kg/m^2^, *p* < 0.001), systolic blood pressure (control: 128.57 mmHg vs. case: 124. 59 mmHg, *p* = 0.049), TC (control: 4.55 mmol/L vs. case: 4.91 mmol/L, *p* = 0.013), TG (control: 0.94 mmol/L vs. case: 2.04 mmol/L, *p* < 0.001), and HDL-C (control: 1.63 mmol/L vs. case: 1.01 mmol/L, *p* < 0.001) levels, as well as the proportion of women (control: 48.44% vs. case: 72.71%, *p* < 0.001). See Table 1 for further details.

The control and case groups used for the HDL subfractions’ analyses were significantly different in mean BMI (control: 24.06 kg/m^2^ vs. case: 28.80 kg/m^2^, *p* < 0.001), TC (control: 4.38 mmol/L vs. case: 4.80 mmol/L, *p* = 0.001), TG (control: 0.90 mmol/L vs. case: 1.95 mmol/L, *p* < 0.001), and HDL-C levels (control: 1.60 mmol/L vs. case: 1.01 mmol/L, *p* < 0.001). In addition, the proportion of women was significantly higher in the case population than in the control population (48.96% vs. 73.33%, *p* < 0.001). See Table 2 for further details.

**Figure 1:**
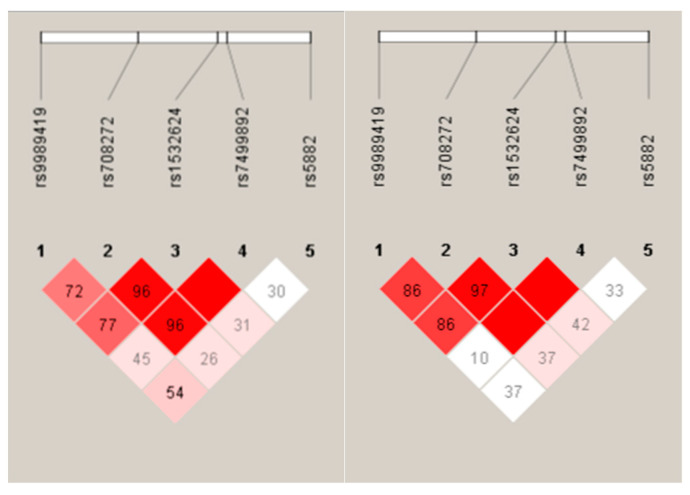
Linkage disequilibrium (LD) map (based on D-prime–D’) of single-nucleotide polymorphisms in the *CETP* gene in the control (**left**) and case (**right**) group. Dark red indicates high LD (D′ = 1 and log of the odds–LOD > 2). Lower LD values are indicated by pink shades (0.21 < D′ < 1 and LOD > 2). White indicates low LD and low LOD (LOD < 2). The number within each box indicates the D statistic value between the corresponding two SNPs.

No significant difference from HWE was observed. The LD maps of the control and case groups are shown in Figure 1. For data on allele frequencies of the five SNPs and the prevalence of their haplotypes per analyses, see Table 3.

### 2.2. Association of CETP Gene Polymorphisms and Their Haplotypes with the Estimated Cardiovascular Risk by SCORE and FRSs

None of the five SNPs examined (rs1532624, rs5882, rs708272, rs7499892, and rs9989419) showed an individually significant association with CVR by SCORE (regardless of the used statistical model). In models I and II, rs7499892 showed a significant association with increased CVR estimated by the FRS_CVR_. In model III, none of the SNPs showed a significant association with CVR after test correction. See Table 4 for further details. 

The H5 showed a significant association with both SCORE and FRSs-estimated CVR in the model I, with FRSs in model II, and no significant association in model III. The H7 showed a significant association with Framingham Risk Score for Coronary Heart Disease (FRS_CHD_) in all three models. The H8 showed a significant association with CVR estimated by SCORE and FRSs in Models I and II. Furthermore, it showed a significant association with Framingham Risk Score for Cardiovascular Disease (FRS_CVD_) in model III. See Table 5 for further details.

### 2.3. Effect of SNPs and Haplotypes Significantly Associated with CVR on TG and HDL-C Levels and HDL Subfraction Profile

None of the SNPs tested showed a significant association with TG levels while rs1532624, rs708272, and rs9989419 showed a significant association with lower HDL cholesterol levels. The C allele of rs1532624 showed a significant negative association with HDL-5 and HDL-I; the G allele of rs708272 with HDL-5, 6, and HDL-I; and the A allele of rs9989419 with HDL-2, 4, 5, 6, and HDL-L and -I. SNPs rs5882 and rs7499892 showed no significant association with TG, HDL-C, or any HDL subfraction. See Supplementary Table S1 for further details.

Among the identified haplotypes, H5 (CAGTA) showed a significant positive association with TG and a negative association with total HDL-C level due to significantly reducing the levels of HDL-6, 7, and 8 subfractions. H7 (AAACA) and H8 (CGGTG) showed no significant association with TG level, total HDL-C level, or HDL subfractions. Further details can be found in Table 6.

## 3. Discussion

This study aimed to investigate the association of five SNPs in the *CETP* gene and their haplotypes with cardiovascular risk estimated by SCORE and FRS_CHD_ and FRS_CVD_.

None of the five tested SNPs showed an individually significant association with SCORE-estimated CVR. For the FRSs, rs7499892 showed a significant association with increased risk in models I and II. No significant association was observed in model III, suggesting that rs7499892 influences CVR through its effect on TG levels (β = 0.176, *p* = 0.027). Previous studies have not found a significant association between rs7499892 and cardiovascular risk or morbidity. A study in a Chinese population [32] found an association between the G allele of rs708272 and an increased risk of coronary atherosclerosis, which is consistent with our results that rs708272 is associated with increased cardiovascular risk estimated by both FRS algorithms (*p* < 0.05), but these were not significant after *p*-value adjustment (*p* < 0.01).

Three haplotypes (H5, H7, and H8) were identified that showed a significant correlation with at least one of the risk estimation algorithms. Our results suggest that the association between H5 (CAGTA) and SCORE-estimated CVR is mediated by its effect on HDL-C level, whereas its association with FRSs is mediated by its combined effect on HDL-C and TG levels. These results are consistent with our previous findings [30] that the H5 haplotype significantly increased the TG/HDL-C ratio, which is associated with increased CVR.

The H8 shows a significant correlation with both SCORE- and FRSs-estimated elevated CVR for models I and II. After correction for TG level (Model III), the result remained significant only for FRS_CVD_. This suggests that the significant association of H8 with SCORE and FRS_CHD_ is through its effect on TG level, whereas in the case of FRS_CVD_, the effect is independent of its impact on HDL-C and TG levels. Based on our previous results [33], H8 is significantly associated with both reduced HDL-C and TG levels but does not significantly affect the TG/HDL-C ratio.

The H7 (AAACA) showed a significant positive association with FRS_CHD_-estimated CVR in all three models in the present study. It did not show a significant association with TG or HDL-C levels (which is consistent with the results of our previous study [33]), or with any of the HDL subfractions. The effect of H7 in increasing the risk of cardiovascular disease is independent of the lipid parameters that we have tested.

Previous studies have shown the effect of SNPs and haplotypes in the *CETP* gene on lipid profile [34,35], interaction with lipid-lowering drugs [36,37], and their impact on the risk of developing CVDs [38,39]. The *CETP* gene and the protein coded are good targets for drug development, which is currently underway. Although the first generation of CETP inhibitors (e.g., torcetrapib [40] and dalcetrapib [41]) mainly raised HDL-C or had off-target effects, and the next generation (e.g., anacetrapib [42] and evacetrapib [43]) have also been shown to be effective in reducing LDL-C, and apoB levels. Anacetrapib was the first CETP inhibitor shown to be effective in reducing the risk of atherosclerotic cardiovascular disease [44]. In addition, CETP inhibitors have been shown to reduce the risk of new-onset diabetes, and improve glucose tolerance and insulin sensitivity [45]. The latest generation of CETP inhibitor obicetrapib, specifically designed to reduce LDL-C and apoB, has achieved significant LDL-C reductions of up to 45% and may be the first CETP inhibitor to serve as adjunctive therapy for patients who do not achieve target LDL-C levels [46]. 

In addition to its direct effects on the lipid profile, previous studies have linked the *CETP* gene and protein to effects on inflammation [47,48], oxidative stress [49,50,51], blood pressure [52], and blood coagulation [53,54,55]. These mechanisms may partly explain the cardiovascular risk-increasing effect of H7 and H8, independent of TG and HDL-C levels.

Our current study had its limitations. Some factors that were not considered in the present study (epigenetic factors, rare or structural variants, gene–gene, and/or gene–environment interactions) also influence the outcomes that we investigated and could alter the results. The present analyses were adjusted for relevant covariates; however, several environmental and lifestyle factors (such as physical inactivity and unhealthy diet) may modify susceptibility to the trait. The cardiovascular risk estimation models used in this study include only a limited number of traditional cardiovascular risk factors (age, sex, smoking, diabetes, blood pressure, and cholesterol) and do not include all known risk factors. A major limitation of the present study is the small sample size, which may result in limited statistical power. Although our results are statistically significant even after Bonferroni correction, it would be useful to perform further analysis in a larger sample of different ethnicities to confirm our findings. Furthermore, since the present study is a cross-sectional one, findings obtained can be considered as single points in time, i.e., longitudinal studies are needed to verify the long-term effects on CVR of the haplotypes identified.

In conclusion, the present study confirms the effect of *CETP* gene polymorphisms and haplotypes on TG level, HDL-C level, and HDL subfraction profile. In this study it has been successfully demonstrated in an independent sample population that the previously identified H5 haplotype is associated with increased CVR and that this effect is mediated by its effect on the TG and HDL-C levels. Two additional haplotypes (H7 and H8) have also been identified that are significantly associated with increased cardiovascular risk, but their effects are partially independent of TG and HDL-C levels and might be mediated by other mechanisms (such as inflammation, blood pressure, blood coagulation) associated with *CETP* gene activity.

## 4. Materials and Methods

### 4.1. Study Design and Populations

A full and more detailed description of the study design and data collection was described in our previous paper [56]. In brief, to understand the background of the very poor health status of the Roma population compared to the Hungarian general one, with a special emphasis on the high prevalence of cardiometabolic diseases, a complex health survey was designed and carried out to create a complex database for association and comparative analyses. The study is based on a cross-sectional survey consisting of three main components: a questionnaire-based survey, physical examinations, and laboratory tests among the adult Hungarian general (HG) and Roma populations aged between 20 and 64 years. The main part of the questionnaire in the survey was the European Health Interview Survey (EHIS) Wave 2 questionnaire (EHIS 2 for 2013–2015, used in the Hungarian survey in 2014), which consists of 4 modules on health status, health care use, health determinants and socioeconomic variables [57]. These modules cover the following topics: self-perceived health status, chronic conditions known to the respondent, activity limitation and mental health, use of different types of health services including hospitalisation, consultations, preventive services and medicines, and unmet health needs, as well as smoking and alcohol consumption, physical activity and dietary habits, and additional background variables on demographic and socioeconomic status such as gender, age, living conditions, education, income, and employment.

A total of 832 participants, including 417 HG (185 men and 232 women) and 415 Roma (108 men and 307 women), were recruited during the survey period (2018/2019). Fasting blood samples were collected for routine laboratory tests (including total cholesterol, TG, LDL cholesterol, HDL-C, and fasting blood glucose measurements), as well as anthropometric (e.g., weight and height), demographic (e.g., sex and age), socioeconomic, and health (including blood pressure measurements and medication used) data were collected.

In the present study, participants with missing anthropometric and/or laboratory parameters (20 HGs and 47 Roma) and participants on lipid-lowering therapy (27 HG and 43 Roma) were excluded from further analysis. The remaining 695 subjects (370 HG and 325 Roma) were divided into two subgroups based on their lipid profile (participants with normal lipid profiles and those with reduced HDL-C). The normal lipid profile group included subjects with normal HDL-C levels (≥1.03 mmol/L in men and ≥1.29 mmol/L in women) and normal levels of TG, TC, and LDL-C (126 HG and 87 Roma). In total, 100 people (25 HG men, 25 Roma men, 25 HG women and 25 Roma women) were randomly selected to form the control group of this study. The second group included all people with reduced HDL-C levels (115 HG and 162 Roma). The HDL subfractions were determined for the 377 individuals selected.

Individuals with incomplete genotype (for five SNPs in the *CETP* gene) or phenotype data were excluded from further analysis. In the present study, three different types of analyses (HDL subfraction profiling, SCORE, and FRS calculations) were performed, with sample numbers varying according to the age criteria.

The analysis of the effect of SNPs and their haplotypes in the *CETP* gene on the HDL subfraction profile was performed on individuals with complete geno- and phenotypic data without age restrictions. These analyses were performed on 96 control and 207 case subjects (4 controls and 7 cases were excluded due to incomplete genotype data).

**Figure 2:**
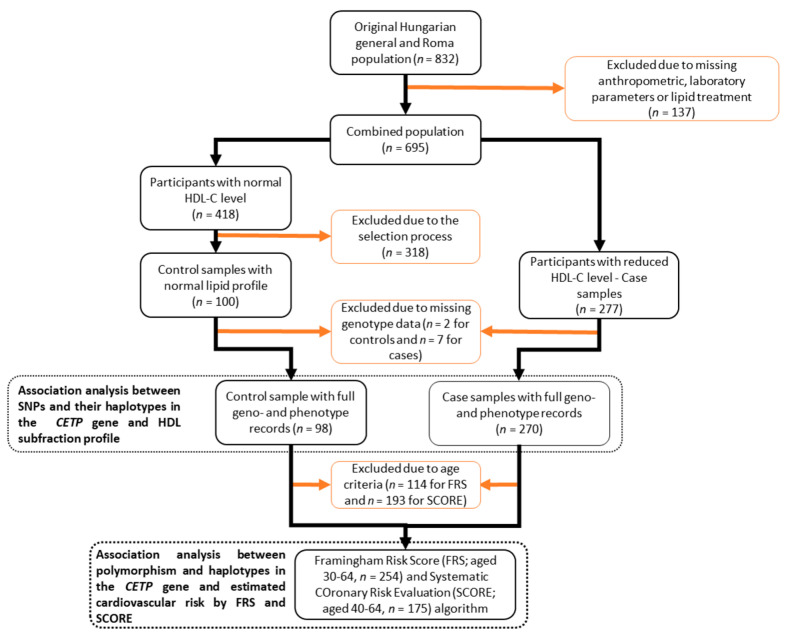
Flowchart showing the process of sample selection to biostatistical analyses on genotype data and risk scores’ calculations.

Analysis of the association of SNPs and their haplotypes with estimated cardiovascular risk was performed on samples of individuals aged 40 to 64 years (52 controls and 123 cases) for SCORE, and 30 to 64 years (64 controls and 191 cases) for FRS due to age restrictions in the application of the algorithms. See Figure 2 for further details.

### 4.2. Analysis of HDL Subfractions

HDL identified solely on the hydration density of its particles is a highly heterogeneous class of lipoproteins. Several methods are known to separate HDL into subfractions. Most of the published prospective and clinical studies have used one of the proprietary laboratory tests or in-house systems available to clinicians to evaluate the use of HDL subfractions to predict outcomes: Lipoprint HDL^®^ (gel electrophoresis), Cardio IQ^®^ (ion mobility), NMR LipoProfile^®^ (nuclear magnetic resonance) and, until recently, Vertical AutoProfile (VAP)^®^ (ultracentrifugation) [58].

For the present study, the Lipoprint HDL Subfractions Test (Quantimetrix Corp., Redondo Beach, CA, USA) was used to determine HDL subfractions according to the manufacturer’s instructions using an electrophoretic method on a polyacrylamide gel. This commercially available test is based on the method of linear polyacrylamide gel electrophoresis and allows the separation and quantification of up to 10 HDL subfractions in serum or plasma.

Briefly, 25 μL of serum was added to the 3% polyacrylamide gel tubes together with 300 μL of Lipoprint HDL Loading Gel solution. The tubes contained Sudan Black as a lipophilic dye and were photopolymerised for 30 min at room temperature. Electrophoresis was performed with tubes containing serum samples and manufacturer’s quality control at a constant current of 3 mA/tube for 50 min. Subfraction bands were identified by their mobility (Rf) using very-LDL (VLDL) + LDL as the start (Rf 0.0) and albumin as the end (Rf 1.0) reference point and were scanned using an ArtixScan M1 digital scanner (Microtek International Inc., Santa Fe Springs, CA, USA).

Ten subfractions of HDL were distinguished between the peaks of VLDL + LDL and albumin and grouped into three main classes: HDL-L (from HDL–1 to 3), HDL-I (from HDL–4 to 7) and HDL-S (from HDL–8 to 10) HDL subfractions. Cholesterol concentrations of the HDL particle subsets were calculated using Lipoware software (version 3.4, Quantimetrix Corp., Redondo Beach, CA, USA). The total cholesterol concentration of the samples was multiplied by the relative area under the curve of the subfraction bands.

### 4.3. Estimation of the Cardiovascular Risk by FRS and SCORE in Study Populations

In the present study, we estimated cardiovascular risk using the two most widely used risk assessment models in Europe (FRS and SCORE). Both algorithms are sex-specific and estimate the risk of a cardiovascular event occurring within 10 years.

The first version of the FRS was developed using data obtained from the Framingham Heart Study [59] to estimate the 10-year risk of developing coronary heart disease (CHD) and was later revised to also calculate the development of CVD in general. In the present study, we used both versions of the FRS developed for hard coronary heart disease [60] and cardiovascular disease [6]. Both versions consider age, sex, total cholesterol, HDL-C levels, systolic blood pressure, treatment for high blood pressure, and current smoking status. In addition to these factors, the FRS_CVD_ algorithm includes the presence of diabetes in the risk estimate. Analyses for the FRSs were performed on participants in the study populations aged 30–64 years.

An estimated risk based on SCORE, which is the algorithm recommended by the 2007 European Society of Cardiology guidelines on cardiovascular disease prevention in clinical practice [7] was also calculated. The model was calibrated according to the mortality statistics of each European country. In the present study, the SCORE algorithm for high-risk countries was applied (due to the Hungarian origin of the samples). All analyses for SCORE were performed on participants in the study populations aged 40–64 years.

A more detailed explanation of the cardiovascular risk models used in this study is described in our previous publication [61].

### 4.4. DNA Isolation, Selection of SNPs, and Genotyping

DNA was isolated using a MagNA Pure LC system (Roche Diagnostics, Basel, Switzerland) with a MagNA Pure LC DNA Isolation Kit-Large according to the manufacturer’s instructions. The extracted DNA was eluted in 200 µL of elution buffer from the MagNA Pure LC DNA Isolation Kit-Large Volume. Genotyping was performed by the Mutation Analysis Core Facility (MAF) at Karolinska University Hospital, Sweden. Genotyping was performed on a MassARRAY platform (Sequenom Inc., San Diego, CA, USA) using the iPLEX Gold chemistry. Validation, concordance analysis, and quality control were performed by the MAF according to their protocols.

The SNPs in the present study were selected based on a systematic literature search to identify polymorphisms significantly associated with HDL-C level in different populations [28]. Based on the results of our previous studies [29,30], the five SNPs and their haplotypes investigated in the present study have been shown to be significantly associated with HDL-C levels independently of ethnicity (HG or Roma).

Of the five SNPs, four are intron variants (rs708272, rs1532624, rs7499892, and rs5882), while one (rs9989419) is located in the regulator region. The LDlink [62] online tool (version 5.6.3, National Institutes of Health (NIH), National Cancer Institute (NCI), Bethesda, MD, USA) was used to determine the localisation (Supplementary Figure S1) and relative LD (Supplementary Table S2) of SNPs (for all populations) using the Genome Reference Consortium Human Build 38 (CRCh38) database.

### 4.5. Statistical Analyses

Statistical tests were performed using the SNPStats online tool [63] (http://bioinfo.iconcologia.net/SNPstats, accessed on 25 April 2023) and IBM SPSS (version 26, IBM Company, Armonk, NY, USA). The linkage disequilibrium (LD) structure was constructed using the Haploview software (version 4.2). The Mann–Whitney U test was used to compare average age, body mass index (BMI), fasting glucose, and HDL-C levels between populations. The prevalence of current smokers, the existence of Hardy–Weinberg equilibrium (HWE), and the differences in allele frequencies for all SNP were assessed using the χ2 test.

The expectation-maximisation algorithm performed by the SNPStats online tool was used to estimate the haplotype block analyses of the SNPs. To avoid effects due to ethnicity-related factors (e.g., environment and culture), the two populations (Hungarian general and Roma) were analysed together, and ethnicity was used as a covariate in the statistical models. To avoid errors due to multicollinearity, all models were adjusted for relevant covariates (e.g., ethnicity, age, sex, BMI, current smoking status, and fasting glucose). Three linear regression models were used to investigate the association of SNPs and haplotypes in the *CETP* gene with SCORE and FRS-estimated CVD risk:

Model I: ethnicity, sex, age, BMI, current smoking status, and fasting glucose levels.

Model II: ethnicity, sex, age, BMI, current smoking status, fasting glucose, and HDL-C levels.

Model III: ethnicity, sex, age, BMI, current smoking status and fasting glucose, HDL-C, and TG levels.

The conventional *p*-value threshold of 0.05 was accepted as significant. A Bonferroni correction analysis was applied where multiple modelling calculations were required to determine the *p*-value cut-off for significance (conventional *p* < 0.05 divided by the number of independent factors). After correction, *p* < 0.005 was considered significant for analyses of HDL subfractions and *p* < 0.01 for analyses of individual effects of SNPs.

## Figures and Tables

**Table 1:** Characteristics of the control and case samples in Systematic Coronary Risk Evaluation (SCORE)—A; Framingham Risk Score for Coronary Heart Disease (FRS_CHD_) and Framingham Risk Score for Cardiovascular Disease (FRS_CVD_) algorithm analyses—B.

**A**	**Control (*n* = 52)**	**Case (*n* = 124)**	***p*** **-Value**
	**Average (Std. Dev.)**		
Age (years)	51.23 (0.90)	49.66 (0.59)	0.164
BMI (kg/m^2^)	25.14 (0.77)	29.29 (0.55)	<0.001 *
Systolic blood pressure (mmHg)	130.10 (2.40)	127.15 (1.46)	0.214
Fasting glucose (mmol/L)	5.55 (0.29)	5.47 (0.15)	0.844
Total cholesterol (mmol/L)	4.59 (0.09)	5.05 (0.10)	0.004 *
Triacylglycerol (mmol/L)	0.97 (0.04)	2.08 (0.10)	<0.001 *
High-density lipoprotein Cholesterol (mmol/L)	1.62 (0.05)	1.01 (0.02)	<0.001 *
	Prevalence in % (95%CI)		*p*-value
Roma	50.00 (36.72–63.28)	54.03 (45.25–62.63)	0.625
Women	48.08 (34.91–61.45)	75.81 (67.73–82.69)	<0.001 *
Current smoker	49.02 (35.67–62.48)	55.28 (46.46–63.86)	0.451
Treated for high blood pressure	28.85 (17.92–42.05)	36.29 (28.22–44.99)	0.342
Treated for diabetes	11.54 (4.96–22.24)	10.48 (6.01–16.78)	0.837
**B**	**Control (*n* = 64)**	**Case (*n* = 191)**	***p*** **-Value**
	**Average (Std. Dev.)**		
Age (years)	48.12 (1.10)	44.29 (0.67)	0.004 *
BMI (kg/m^2^)	24.78 (0.67)	29.07 (0.45)	<0.001 *
Systolic blood pressure (mmHg)	128.57 (2.08)	124.59 (1.14)	0.049 *
Fasting glucose (mmol/L)	5.28 (0.25)	5.26 (0.11)	0.950
Total cholesterol (mmol/L)	4.55 (0.08)	4.91 (0.08)	0.013 *
Triacylglycerol (mmol/L)	0.94 (0.04)	2.04 (0.08)	<0.001 *
High-density lipoprotein cholesterol (mmol/L)	1.63 (0.05)	1.01 (0.01)	<0.001 *
	Prevalence in % (95%CI)		*p*-value
Roma	50.00 (37.98–62.02)	45.03 (38.09–52.11)	0.490
Women	48.44 (36.49–60.52)	72.77 (66.15–78.71)	<0.001 *
Current smoker	49.21 (37.13–61.35)	56.84 (49.74–63.74)	0.291
Treated for high blood pressure	25.00 (15.65–36.55)	30.89 (24.66–37.69)	0.371
Treated for diabetes	9.38 (4.01–18.30)	7.85 (4.66–12.31)	0.702

*: *p* < 0.05.

**Table 2:** Characteristics of the control and case samples used for HDL subfraction profile analyses.

	Control (*n* = 96)	Case (*n* = 270)	*p*-Value
	Average (Std. Dev.)		
Age (years)	41.82 (1.34)	40.45 (0.72)	0.382
BMI (kg/m^2^)	24.06 (0.53)	28.80 (0.37)	<0.001 *
Systolic blood pressure (mmHg)	124.85 (1.71)	123.02 (0.94)	0.272
Fasting glucose (mmol/L)	5.09 (0.19)	5.23 (0.10)	0.381
Total cholesterol (mmol/L)	4.38 (0.07)	4.80 (0.07)	0.001 *
Triacylglycerol (mmol/L)	0.90 (0.03)	1.95 (0.07)	<0.001 *
High-density lipoprotein cholesterol (mmol/L)	1.60 (0.04)	1.01 (0.01)	<0.001 *
	Prevalence in % (95%CI)		*p*-value
Roma	51.04 (41.14–60.89)	58.52 (52.58–64.28)	0.204
Women	48.96 (39.11–58.86)	73.33 (67.83–78.34)	<0.001 *
Current smoker	48.42 (38.55–58.39)	57.25 (51.29–63.06)	0.137
Treated for high blood pressure	20.83 (13.65–29.75)	27.41 (22.35–32.95)	0.205
Treated for diabetes	7.29 (3.32–13.78)	7.04 (4.44–10.55)	0.934

*: *p* < 0.05.

**Table 3:** The distribution of minor and major allele frequencies of the five SNPs and their haplotypes by Systematic Coronary Risk Evaluation (SCORE), Framingham Risk Score for Hard Coronary Heart Disease (FRS_CHD_) and Cardiovascular Disease (FRS_CVD_) and HDL subfraction profiles.

	SCORE (*n* = 176)	FRS_CHD_ and FRS_CVD_ (*n* = 255)	HDL Subfractions’ Profile (*n* = 366)
SNPs (minor/major allele)	Frequency in %		
rs1532624 (A/C)	27.27/72.73	29.71/70.29	30.33/69.67
rs5882 (G/A)	28.69/71.31	28.82/71.18	28.76/71.24
rs708272 (A/G)	29.26/70.74	31.37/68.63	31.76/68.24
rs7499892 (T/C)	36.08/63.92	33.34/66.66	33.74/66.26
rs9989419 (G/A)	46.45/53.55	47.06/52.94	48.84/51.16
Haplotypes (H)	Prevalence in %		
H1 (AGACG)	22.29	22.27	23.13
H2 (AAACG)	13.76	14.50	14.74
H3 (CAGCA)	14.79	15.73	14.04
H4 (CAGCG)	8.86	8.46	9.03
H5 (CAGTA)	16.79	14.90	15.90
H6 (CGGCA)	5.26	5.67	6.20
H7 (AAACA)	2.37	2.87	2.76
H8 (CGGTG)	5.65	5.22	4.78
H9 (CGGCG)	3.25	3.49	3.14
H10 (CAGTG)	2.72	2.38	2.84

**Table 4:** Association of SNPs in the *CETP* gene with cardiovascular risk estimated by Systematic Coronary Risk Evaluation (SCORE) and Framingham Risk Score for Hard Coronary Heart Disease (FRS_CHD_) and Cardiovascular Disease (FRS_CVD_). The analyses were adjusted in model I for ethnicity, sex, age, BMI, current smoking status, and fasting glucose levels; in model II for ethnicity, sex, age, BMI, current smoking status, fasting glucose, and HDL-C levels; and in model III for ethnicity, sex, age, BMI, current smoking status and fasting glucose, HDL-C, and TG levels. The effect allele of SNPs is shown in brackets.

**Model I.**	**SCORE**		**FRS_CHD_**		**FRS_CVD_**	
	**β (Std. Dev.)**	***p*** **-Value**	**β (Std. Dev.)**	***p*** **-Value**	**β (Std. Dev.)**	***p*** **-Value**
rs1532624 (C allele)	0.190 (0.130)	0.144	0.445 (0.205)	0.031 *	0.916 (0.384)	0.018 *
rs5882 (A allele)	0.141 (0.120)	0.243	0.318 (0.201)	0.116	0.442 (0.381)	0.248
rs708272 (G allele)	0.159 (0.124)	0.201	0.467 (0.199)	0.020 *	0.916 (0.372)	0.014 *
rs7499892 (T allele)	0.260 (0.143)	0.070	0.774 (0.250)	0.002 **	1.580 (0.467)	<0.001 **
rs9989419 (A allele)	0.133 (0.118)	0.262	0.276 (0.195)	0.159	0.308 (0.368)	0.403
**Model II.**	**SCORE**		**FRS_CHD_**		**FRS_CVD_**	
	**β (std. dev.)**	***p*** **-value**	**β (std. dev.)**	***p*** **-value**	**β (std. dev.)**	***p*** **-value**
rs1532624 (C allele)	0.090 (0.123)	0.467	0.357 (0.203)	0.080	0.641 0.376)	0.089
rs5882 (A allele)	0.049 (0.113)	0.669	0.269 (0.198)	0.175	0.262 (0.368)	0.477
rs708272 (G allele)	0.067 (0.117)	0.571	0.389 (0.197)	0.050 *	0.671 (0.363)	0.066
rs7499892 (T allele)	0.192 (0.133)	0.151	0.693 (0.246)	0.005 **	1.344 (0.452)	0.003 **
rs9989419 (A allele)	0.031 (0.111)	0.780	0.136 (0.195)	0.488	−0.067 (0.361)	0.853
**Model III.**	**SCORE**		**FRS_CHD_**		**FRS_CVD_**	
	**β (std. dev.)**	***p*** **-value**	**β (std. dev.)**	***p*** **-value**	**β (std. dev.)**	***p*** **-value**
rs1532624 (C allele)	0.061 (0.122)	0.616	0.320 (0.184)	0.084	0.581 (0.350)	0.098
rs5882 (A allele)	0.043 (0.111)	0.700	0.120 (0.181)	0.506	0.018 80.345)	0.960
rs708272 (G allele)	0.055 (0.115)	0.637	0.400 (0.178)	0.026 *	0.694 (0.337)	0.040 *
rs7499892 (T allele)	0.156 (0.132)	0.240	0.454 (0.227)	0.046 *	0.978 (0.428)	0.023 *
rs9989419 (A allele)	0.015 80.109)	0.891	0.121 (0.177)	0.496	−0.091 (0.336)	0.787

*: *p* < 0.05 (conventional *p*-value), **: *p* < 0.01 (Bonferroni corrected *p*-value).

**Table 5:** Association of haplotypes with estimated cardiovascular risk according to the Systematic Coronary Risk Evaluation (SCORE), Framingham Risk Score for Hard Coronary Heart Disease (FRS_CHD_), and Framingham Risk Score for Cardiovascular Disease (FRS_CVD_) algorithms. Model I adjusted for age, sex, ethnicity, BMI, current smoking status, and fasting glucose levels; model II adjusted for HDL-C levels in addition to the above; and model III adjusted for TG levels in addition to the above.

	H1	H2	H3	H4	H5	H6	H7	H8	H9	H10
rs1532624	A	A	C	C	C	C	A	C	C	C
rs5882	G	A	A	A	A	G	A	G	G	A
rs708272	A	A	G	G	G	G	A	G	G	G
rs7499892	C	C	C	C	T	C	C	T	C	T
rs9989419	G	G	A	G	A	A	A	G	G	G
Model I.	β (95%CI), *p*-value									
SCORE	Ref.	N.S.	N.S.	N.S.	0.43 (0.05–0.81) *p* = 0.028 *	N.S.	N.S.	0.82 (0.18–1.46) *p* = 0.012 *	N.S.	N.S.
FRS_CHD_		N.S.	N.S.	N.S.	0.98 (0.30- 1.64) *p* = 0.003 *	N.S.	1.79 (0.27–3.31) p = 0.022 *	1.48 (0.30–2.66) *p* = 0.014 *	N.S.	N.S.
FRS_CVD_		N.S.	N.S.	N.S.	1.93 (0.69–3.16) *p* = 0.002 *	N.S.	N.S.	3.04 (0.92–5.17) *p* = 0.005 *	N.S.	N.S.
Model II.	Β (95%CI), *p*-value									
SCORE	Ref.	N.S.	N.S.	N.S.	N.S.	N.S.	N.S.	0.62 (0.01–1.24) *p* = 0.047 *	N.S.	N.S.
FRS_CHD_		N.S.	N.S.	N.S.	0.80 (0.14–1.45) *p* = 0.017 *	N.S.	1.63 (0.18–3.09) p = 0.028 *	1.27 (0.11–2.44) *p* = 0.033 *	N.S.	N.S.
FRS_CVD_		N.S.	N.S.	N.S.	1.46 (0.25–2.66) *p* = 0.018 *	N.S.	N.S.	2.97 (1.00–4.94) *p* = 0.003 *	N.S.	N.S.
Model III	β (95%CI), *p*-value									
SCORE	Ref.	N.S.	N.S.	N.S.	N.S.	N.S.	N.S.	N.S.	N.S.	N.S.
FRS_CHD_		N.S.	N.S.	N.S.	N.S.	N.S.	1.43 (0.15–2.71) *p* = 0.029 *	N.S.	N.S.	N.S.
FRS_CVD_		N.S.	N.S.	N.S.	N.S.	N.S.	N.S.	2.29 (0.29–4.28) *p* = 0.025 *	N.S.	N.S.

N.S., non-significant; *, *p* < 0.05 (conventional *p*-value).

**Table 6:** Effect of haplotypes (H) significantly associated with triacylglycerol (TG), high-density lipoprotein cholesterol (HDL-C) levels, and HDL subfractions (in mmol/L). All analyses were adjusted for ethnicity, sex, age, BMI, fasting glucose levels, and current smoking status.

	H1	H5	H7	H8
rs1532624	A	C	A	C
rs5882	G	A	A	G
rs708272	A	G	A	G
rs7499892	C	T	C	T
rs9989419	G	A	A	G
		β (95%CI)		
TG	Ref.	0.43 (0.16–0.69) *p* = 0.002 **	N.S.	N.S.
HDL-C	Ref.	−0.12 (−0.20–−0.04), *p* = 0.004 **	N.S.	N.S.
HDL-1	Ref.	N.S.	N.S.	−0.02 (−0.04–−0.00) *p* = 0.030 *
HDL-2		N.S.	N.S.	N.S.
HDL-3		N.S.	−0.04 (−0.08–−0.01) *p* = 0.024 *	N.S.
HDL-4		N.S.	N.S.	N.S.
HDL-5		N.S.	−0.01 (−0.02–0.00) *p* = 0.020 *	N.S.
HDL-6		−0.03 (−0.04–−0.01) *p* = 0.003 **	N.S.	N.S.
HDL-7		−0.01 (−0.02–0.00) *p* = 0.002 **	N.S.	−0.01 (−0.03–0.00) *p* = 0.019 *
HDL-8		−0.01 (−0.02–0.00) *p* = 0.001 **	N.S.	−0.01 (−0.02–0.00) *p* = 0.012 *
HDL-9		−0.01 (−0.01–0.00) *p* = 0.019 *	N.S.	N.S.
HDL-10		N.S.	N.S.	N.S.
Large HDL	Ref.	N.S.	N.S.	N.S.
Interm. HDL		−0.05 (−0.09–−0.02) *p* = 0.008 *	N.S.	N.S.
Small HDL		N.S.	N.S.	N.S.

N.S., non-significant; Interm., intermediate; *, *p* < 0.05 (conventional *p*-value); **, *p* < 0.005 (Bonferroni corrected *p*-value).

## Data Availability

Data are available on request due to privacy or ethical concerns.

## Supplementary Materials

The following supporting information can be downloaded at: https://www.mdpi.com/article/10.3390/ijms241210281/s1.
