# Supplementary material for: Association of CETP Gene Polymorphisms and Haplotypes with Cardiovascular Risk
Source: Int J Mol Sci. 2023 Jun 17;24(12):10281. doi: 10.3390/ijms241210281 (PMC10299660; doi:10.3390/ijms241210281)
Supplement: Supplementary file 1 [file ijms-24-10281-s001.zip › Sup. figure 1.pdf]

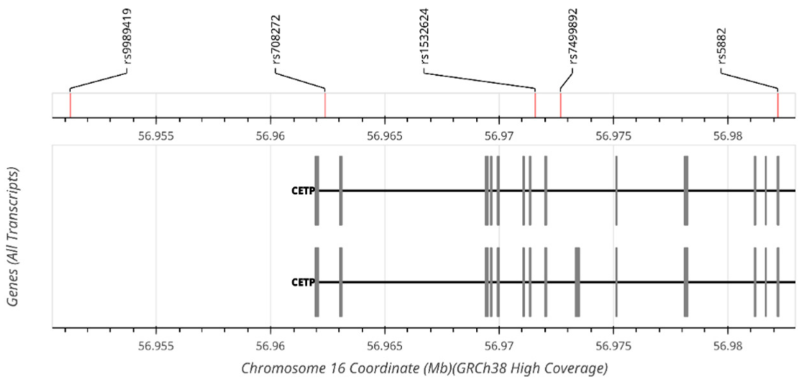

Supplementary Figure S1. Localisation of the five SNPs investigated (rs1532624, rs5882, rs708272, rs7499892, and rs9989419) in the genome as it is showed by using the LDlink online tool on the Genome Reference Consortium Human Build 38 (CRCh38) database.
