# Supplementary material for: Association of CETP Gene Polymorphisms and Haplotypes with Cardiovascular Risk
Source: Int J Mol Sci. 2023 Jun 17;24(12):10281. doi: 10.3390/ijms241210281 (PMC10299660; doi:10.3390/ijms241210281)
Supplement: Supplementary file 1 [file ijms-24-10281-s001.zip › Sup. table 1.pdf]

Supplementary Table S1. Association of SNPs in the *CETP* gene with triacylglycerol (TG), high-density lipoprotein cholesterol (HDL-C) levels and HDL subfractions (in mmol/L).

	rs1532624 – C allele	rs708272 – G allele	rs7499892 – T allele
		β (95% CI)	
TG	N.S.	N.S.	0.176 (0.020 – 0.332) *p* = 0.027*
HDL-C	-0.072 (-0.115 – -0.029) *p* = 0.001**	-0.068 (-0.110 – -0.026) *p* = 0.002**	-0.056 (-0.107 – -0.005) *p* = 0.033*
HDL-1	-0.006 (-0.011 – 0.001) *p* = 0.025*	-0.005 (-0.010 – -0.001) *p* = 0.029*	-0.007 (-0.13 – -0.001) *p* = 0.031*
HDL-2	-0.010 (-0.018 – -0.002) *p* = 0.014*	-0.009 (-0.017 – -0.001) *p* = 0.024*	N.S.
HDL-3	-0.010 (-0.019 – -0.002) *p* = 0.017*	-0.009 (-0.017 – -0.001) *p* = 0.035*	N.S.
HDL-4	-0.010 (-0.017 – -0.002) *p* = 0.012*	-0.009 (-0.016 – -0.001) *p* = 0.022*	N.S.
HDL-5	-0.008 (-0.013 – -0.003) *p* <0.001**	-0.008 (-0.012 – -0.003) *p* = 0.001**	N.S.
HDL-6	-0.011 (-0.019 – -0.003) *p* = 0.007*	-0.011 (-0.019 – -0.004) *p* = 0.005**	-0.011 (-0.021 – -0.001) *p* = 0.027*
HDL-7	-0.004 (-0.007 – 0.000) *p*=0.042*	-0.004 (-0.007 – -0.001) *p* = 0.024*	-0.004 (-0.008 – 0.000) *p* = 0.041*
HDL-8	N.S.	-0.003 (-0.006 – 0.000) *p* = 0.044*	-0.004 (-0.007 – 0.000) *p* = 0.037*
HDL-9	N.S.	N.S.	N.S.
HDL-10	N.S.	N.S.	N.S.
Large HDL	-0.026 (-0.046 – -0.006) *p* = 0.009*	-0.023 (-0.043 – -0.004) *p* = 0.019*	-0.024 (-0.047 – 0.000) *p* = 0.050*
Interm. HDL	-0.034 (-0.055 – -0.012) *p* = 0.002**	-0.033 (-0.054 – -0.012) *p* = 0.002**	-0.027 (-0.052 – -0.002) *p* = 0.036*
Small HDL	N.S.	N.S.	N.S.

N.S.: non-significant; Interm.: intermediate; *: *p* <0.05 (conventional *p*-value); **: *p* <0.005 (Bonferroni corrected *p*-value)
