# Supplementary material for: Association of CETP Gene Polymorphisms and Haplotypes with Cardiovascular Risk
Source: Int J Mol Sci. 2023 Jun 17;24(12):10281. doi: 10.3390/ijms241210281 (PMC10299660; doi:10.3390/ijms241210281)
Supplement: Supplementary file 1 [file ijms-24-10281-s001.zip › Sup. table 2.pdf]

Supplementary Table S2. The results of linkage disequilibrium analyses for the five SNPs (rs1532624, rs5882, rs708272, rs7499892, and rs9989419), as it is, showed by using the LDlink online tool on the Genome Reference Consortium Human Build 38 (CRCh38) database.

	rs9989419	rs7082272	rs1532624	rs7499892
rs7082272	D′: 0.486			
rs1532624	D′: 0.620	D′: 0.812		
rs7499892	D′: 0.462	D′: 0.779	D′: 0.995	
rs5882	D′: 0.091	D′: 0.291	D′: 0.341	D′: 0.343
